# Involvement of Neural Transient Receptor Potential Channels in Peripheral Inflammation

**DOI:** 10.3389/fimmu.2020.590261

**Published:** 2020-10-23

**Authors:** Harold A. Silverman, Adrian Chen, Nigel L. Kravatz, Sangeeta S. Chavan, Eric H. Chang

**Affiliations:** ^1^ Laboratory of Biomedical Science, Feinstein Institutes for Medical Research, Northwell Health, Manhasset, NY, United States; ^2^ Institute of Bioelectronic Medicine, Feinstein Institutes for Medical Research, Northwell Health, Manhasset, NY, United States; ^3^ Donald and Barbara Zucker School of Medicine at Hofstra/Northwell, Hofstra University, Hempstead, NY, United States

**Keywords:** pain, itch, thermal sensing, nervous system, vagus nerve, cytokine

## Abstract

Transient receptor potential (TRP) channels are a superfamily of non-selective cation channels that act as polymodal sensors in many tissues throughout mammalian organisms. In the context of ion channels, they are unique for their broad diversity of activation mechanisms and their cation selectivity. TRP channels are involved in a diverse range of physiological processes including chemical sensing, nociception, and mediating cytokine release. They also play an important role in the regulation of inflammation through sensory function and the release of neuropeptides. In this review, we discuss the functional contribution of a subset of TRP channels (TRPV1, TRPV4, TRPM3, TRPM8, and TRPA1) that are involved in the body’s immune responses, particularly in relation to inflammation. We focus on these five TRP channels because, in addition to being expressed in many somatic cell types, these channels are also expressed on peripheral ganglia and nerves that innervate visceral organs and tissues throughout the body. Activation of these neural TRP channels enables crosstalk between neurons, immune cells, and epithelial cells to regulate a wide range of inflammatory actions. TRP channels act either through direct effects on cation levels or through indirect modulation of intracellular pathways to trigger pro- or anti-inflammatory mechanisms, depending on the inflammatory disease context. The expression of TRP channels on both neural and immune cells has made them an attractive drug target in diseases involving inflammation. Future work in this domain will likely yield important new pathways and therapies for the treatment of a broad range of disorders including colitis, dermatitis, sepsis, asthma, and pain.

## Introduction

**Figure 1:**
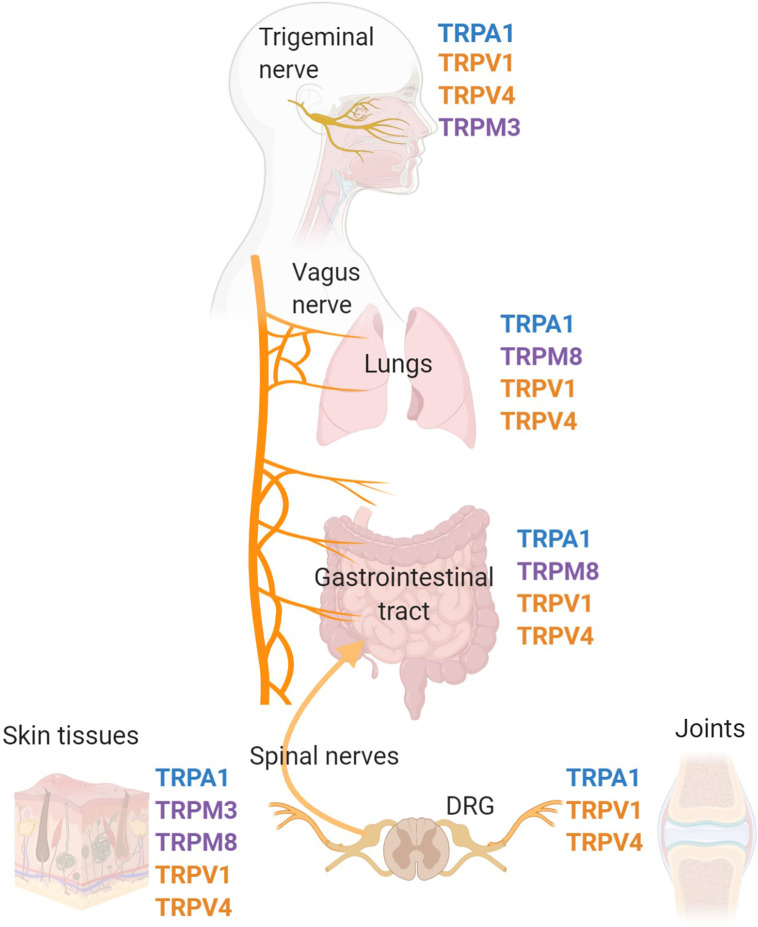
TRP channel-expressing peripheral nerves innervate major organs of the body and effect inflammation. Pre-clinical and clinical studies have identified that the TRP channels, TRPV1, TRPV4, TRPM3, TRPM8, and TRPA1 are expressed on a variety of cell types, specifically sensory nerves that innervate peripheral tissues throughout the body. Major sites of TRP channel expression include the trigeminal nerve, vagus nerve, dorsal root ganglia and associated spinal nerves. The trigeminal nerve is the fifth cranial nerve innervating the face and sinus, with the cell bodies located in the trigeminal ganglia. The vagus nerve is the tenth cranial nerve which innervates many peripheral organs including the lungs and gastrointestinal tract. Spinal nerves, with cell bodies located in the dorsal root ganglia (DRG), innervate many tissues in the periphery including the skin, joints, and colon.

Transient receptor potential (TRP) channels are polymodal calcium-permeable cation channels that broadly act as cellular sensors. Mammalian TRP channels consist of 28 members and can be grouped into six main families: TRP ankrin (TRPA), TRP canonical (TRPC), TRP melastatin (TRPM), TRP mucolipins (TRPML), TRP polycystin (TRPP), and TRP vanilloid (TRPV). For the purposes of this review, we will focus on specific channels within the TRPA, TRPM, and TRPV families that have documented roles and mechanisms relevant to inflammation. There is already an extensive body of literature covering the many different TRP families, their protein structures, and their specific functions, therefore, the goal of this review is to highlight a specific set of TRP channels that are expressed in the peripheral nervous system and have been linked to immune system responses. These TRP channels, specifically TRPA1, TRPM3, TRPM8, TRPV1, and TRPV4 are expressed on peripheral nerves and neurons that communicate with the immune system and major peripheral organs to regulate inflammatory responses (1–10) (**Figure 1**).

We will focus on the broad role of these neural TRP channels as well as their role on neurons and peripheral nerves in mediating the crosstalk between the nervous system and immune system, particularly in the context of inflammation. The activation of TRP channels has an increasingly recognized role in a wide range of inflammatory disorders and therefore may be suitable as potential targets for therapeutic intervention.

## TRP Channel Function and Expression

### TRP “Vanilloid” Channels

The Transient Receptor Potential Vanilloid (TRPV) channel subfamily consists of six members: TRPV1-V6, with TRPV1-4 classified as the thermo-TRPs that are activated by heat in heterologous expression systems (11). TRPV proteins contain seven hydrophobic domains with six spanning the cellular membrane (S1–S6) and the seventh hydrophobic domain, as well as the C- and N- termini, located within the cell (12).

### TRPV1

**Figure 2:**
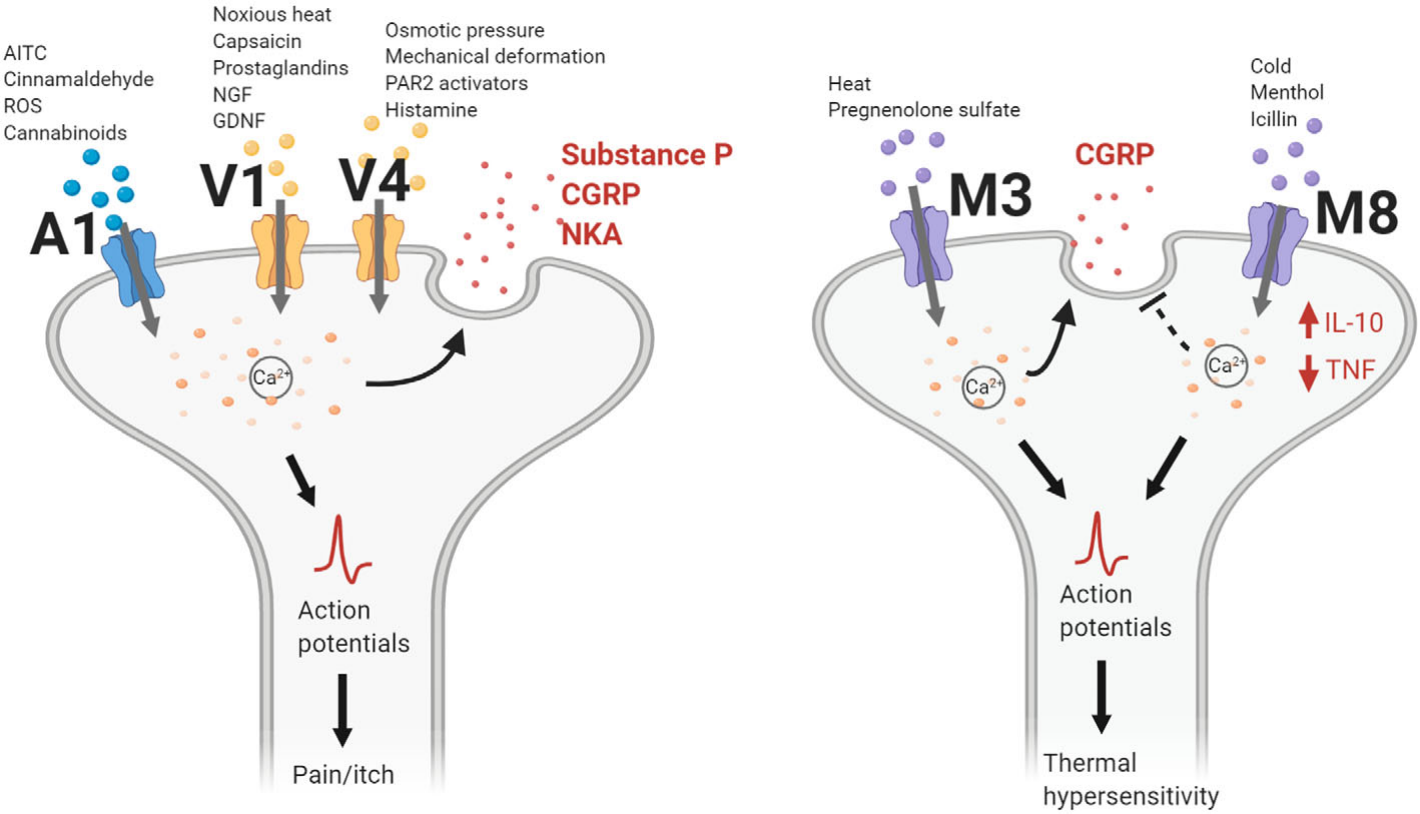
TRP channel actions contributing to neurogenic inflammation. TRPA1, TRPV1, and TRPV4 channels gate cations following activation by their respective chemical agonists, temperature changes, or mechanical stimulation. Intracellular calcium levels increase and lead to the release of neuropeptides such as calcitonin gene-related peptide (CGRP), substance P (SP), or neurokinin A (NKA). Nerve action potentials trigger the sensation of pain or itch. For TRPM3, heat changes and pregnenolone sulfate activate the channel to gate cations, leading to CGRP release. Conversely, TRPM8 activation inhibits CGRP release while increasing levels of interleukin-10 (IL-10) and decreasing levels of tumor necrosis factor (TNF). Action potentials generated following TRPM3/8 channel activation lead to changes in thermal sensitivity. AITC, allyl isothiocyanate; GDNF, glial cell line-derived neurotrophic factor; NGF, nerve growth factor; PAR2, protease-activated receptor 2; ROS, reactive oxidative species.

TRPV1 is a nonselective, calcium permeable, cation channel, and the first member for the TRPV family of ion channels discovered. Activated by a multitude of endogenous and exogenous compounds, TRPV1 is the most extensively studied of the TRPV channels (**Figure 2**) (1). Endogenous endocannabinoids, anandamide (13), N-arachidonoyl-dopamine (14), as well as endogenous lipoxygenase products, 12-(S)-hydroperoxyeicosatetraenoic acid, and leukotriene B4 (15), serve as ligands to TRPV1. Exogenous chemicals such as resiniferatoxin, olvanil, and most prominently, capsaicin, the main irritant found in hot chili peppers (16), also effectively activate TRPV1. In addition to these substances, temperatures greater than 43°C and acidic conditions with a pH lower than 6.0, can induce TRPV1 activation leading to a burning sensation and pain (17–19).

The initial discovery of TRPV1 was through the sequencing of genes expressed in dorsal root ganglion neurons (DRG) (16). Subsequently, it has been reported that the majority of DRG neurons express TRPV1 (2). In addition to DRGs, TRPV1 is highly expressed in nodose ganglia (NG) and trigeminal ganglia (TG), specifically on unmyelinated C- and thinly myelinated Aδ-type sensory nerve fibers (20). In the central nervous system (CNS), TRPV1 is expressed on dopaminergic neurons of the substantia nigra, hippocampal pyramidal neurons, hypothalamic neurons, locus coeruleus neurons, and the cerebral cortex (21). TRPV1 is also found to be expressed on a variety of non-neuronal cell types, including immune cells such as T lymphocytes (22), macrophages (23), and dendritic cells (24). Non-neural and non-immune cell expression of TRPV1 can be found on keratinocytes (25), bladder urothelium (26), smooth muscle (27), hepatocytes (28), pancreatic β-cells (29), and endothelial cells (30).

### TRPV4

TRPV4 is a nonselective, moderately calcium permeable, cation channel. It has a homo-dimeric tetramer structure with the TRPV family standard of six transmembrane segments. The pore loop is between segments 5 and 6 and both the C- and N-termini are located within the cytoplasm (31). The channel contains six ankyrin repeats and the phospholipid, phosphatidylinositol 4,5-bisphosphate (PIP2), can bind to this site inhibiting the channel (32). Similar to TRPV1, TRPV4 is expressed on a variety of cells such as neurons, leukocytes (33), T cells (34), and macrophages (35). In the brain, neurons and glial cells in the hippocampus, cortex, thalamus, cerebellum (36), and hypothalamus (37) all express TRPV4. In the peripheral nervous system, TRPV4 is extensively expressed on DRG, NG, and TG neurons (38, 39) (**Figure 1**). DRG neurons containing this channel are found innervating the spinal dorsal horn (40), gastrointestinal tract (38), skin (41), and liver (42). TRPV4-positive nerve fibers have been also found to innervate arrector pili smooth muscle of the skin, sweat glands, intestines, and blood vessels, and dura mater (39, 43).

### TRP “Melastatin” Channels

The transient receptor potential melastatin (TRPM) subfamily has been regarded as the most diverse group of TRP channels, comprised of eight nonselective cation channels: TRPM1, TRPM2, TRPM3, TRMP4, TRPM5, TRPM6/7, and TRPM8 (3). These channels were first identified as the protein that decreases in expression in highly metastatic melanoma cell lines (44). Structurally akin to those of voltage-gated channels, all TRPM channels possess six transmembrane domains, a TRP helix, and a cytoplasmic N- and C-terminal (45, 46). The widely expressed family of TRPV channels has been discovered to contribute toward a variety of physiological functions from sensing oxidative stress, temperature changes, and cell swelling. While several of these channels play a crucial role in the nervous system (e.g., neuroinflammation), TRPM3 and TRPM8 are the most prominent channels among sensory nerves, particularly in the skin, sinuses, lungs, and the gastrointestinal tract (7, 47–52) (**Figure 1**).

### TRPM3

First discovered through residual heat sensitivity testing in TRPV1 KO mice, the transient receptor potential melastatin-3 (TRPM3) is a non-selective calcium cation channel that has been recently observed to play a crucial role in noxious heat detection (45, 47, 48). TRPM3 is widely expressed in both neuronal and non-neuronal tissue, such as in brain and spinal tissues, retinal (53), pituitary, kidney, and testes (49). Through *in situ* hybridization and RT-qPCR of TRPM3 mRNA, studies have shown abundant expression in both TG and DRG sensory neurons (**Figure 1**) (54). A large majority of TRPM3-expressing neurons are also responsive to capsaicin, demonstrating that it is often co-expressed with TRPV1. The functional detection of TRPM3 has been identified using calcium imaging through chemical activation on DRG cells (45).

### TRPM8

TRPM8 is a nonselective, calcium-permeable cation channel. Among the thermal-sensing TRP channels, the TRPM8 channel is notable for detecting cold temperatures (8–26°C) and contributing to the cooling sensation by chemicals such as menthol and icilin (**Figure 2**). TRPM8 has been found to be expressed on C- and Aδ- sensory nerve fibers, as well as DRG and TG neurons (48). In the CNS, expression of TRPM8 has been found on hypothalamic and hindbrain nuclei responsible for autonomic thermoregulation (55). In addition to neuronal cell types, TRPM8 is also expressed on macrophages and pulmonary epithelial cells. Activation of TRPM8 on macrophages has been shown to induce an anti-inflammatory response with the increased release of interleukin 10 (IL-10) and decreased release of tumor necrosis factor (TNF). In contrast, activation of TRPM8 on pulmonary epithelial cells increases the expression of pro-inflammatory cytokines such as TNF and interleukin 1 (IL-1) (50).

### TRP “Ankyrin” Channels

Transient receptor potential ankyrin 1 (TRPA1) is the only member of the TRPA family. TRPA1 is a polymodal cation channel that is made up of approximately 1,100 amino acids, with roughly 80% of its molecular mass located in the large intracellular domain (56) and a 14 ankyrin repeat in its structure (50, 57). When first described, TRPA1 was reported to sense cold temperatures (<17°); however, it has since been found to additionally sense heat, a common function of TRPV1 and TRPM3 as mentioned above (58–61). Some evidence indicates that the role of TRPA1 as a bidirectional thermo-sensor is due to different channel conformations, and that its heat sensing properties are dependent on its redox state and ligands (60). Along with its role as a thermo-sensor, TRPA1 also responds to mechanical stimuli *via* membrane stress, in a redox state dependent manner (62, 63).TRPA1 is predominantly expressed on myelinated Aδ- and unmyelinated C-fibers of peripheral nerves. Protein expression is found on both cell bodies of DRG, NG, and TG neurons, as well as on the axons of spinal nerves, the vagus nerve, and trigeminal nerve (**Figure 1**) (4, 64). Although not extensively studied, some TRPA1 expression has also been found in regions of the brain such as the somatosensory cortex, and cerebellum (65, 66). Additionally, TRPA1 channels can be found on non-neuronal cell types. TRPA1 was first cloned in fibroblasts (67) and has since been found to be expressed on T-cells, macrophages, endothelial cells, epithelial cells, and smooth muscle cells (50, 68–71). Along with its role as a thermo- and mechanosensory, TRPA1 is activated by a wide variety of chemical stimuli such as cinnamaldehyde, allyl isothiocyanate (AITC), allicin, hydrogen peroxide, oxygen (O_2_), nitroxyl (HNO), methylglyoxal, and endotoxin (lipopolysaccharide; LPS) (**Figure 2**) (4, 72–75). Many of these activators have been found to also play a role in modulating inflammatory responses.

## Neural TRP Channels in the Context of Inflammation

### TRPV1

TRPV1 has been found to be have a key role in inflammation, being linked to both pro- and anti- inflammatory mechanisms. Noxious heat, which can cause cell damage and even death, is a mediator of TRPV1 activation inducing hyperalgesia or pain (**Figure 2**). Increased thermal sensitivity of the TRPV1 channel is mediated by bradykinin and nerve growth factor (NGF) *via* the hydrolysis of intracellular phosphatidylinositol 4,5-bisphosphate (PIP2) (76). After retrograde transport of NGF in peripheral nerves thermal hypersensitivity is maintained *via* changes in TRPV1 expression through the activation of p38, a regulator of pro-inflammatory cytokines TNF, IL-1β, and cyclooxygenase-2 (77). Glial cell line-derived neurotrophic factor (GDNF) family members have also been shown to activate TRPV1 on DRGs, leading to increased thermal hyperalgesia (78). Additionally, protein kinase A (PKA) increases TRPV1’s sensitivity to heat and capsaicin through phosphorylation of TRPV1 on Ser-502 (79, 80). PKA can reduce a desensitized state of TRPV1 through the phosphorylation of Thr-370 and Ser-116, (81). In rats in order for TRPV1 to respond to capsaicin Calmodulin-kinase II must be phosphorylated on Ser-502 and Thr-704 (80). Similarly, protein kinase C (PKC), sensitizes TRPV1 when phosphorylated. This occurs when inflammatory mediators prostaglandin E2 and prostaglandin I2 signal through the prostaglandin EP1 and prostacyclin receptors in a PKC dependent manner (82). Adenosine triphosphate (ATP), released from damaged cells after trauma, also activates and sensitizes these channels through P2Y receptors. In such instances, the threshold temperature for TRPV1 was decreased enough to activate the channel under normal physiological conditions (82, 83). It has been demonstrated that some inflammatory mediators, including TNF, NGF and ATP, promote the recruitment of TRPV1 to the surface of the cell, while other mediators, such as bradykinin and GDNFs, act by decreasing the activation threshold of the channel without affecting expression density (84, 85). Furthermore, additional inflammatory mediators released by macrophages and neutrophils such as reactive nitrogen species (RNS) and reactive oxygen species (ROS) can directly activate TRPV1 on afferent vagus neurons (86). During inflammation, acidic conditions can also be produced within the affected tissue activating TRPV1, leading to TRPV1 sensitization and pain (87, 88).

TRPV1 expression and functionality on neurons is significantly altered depending on the inflammatory condition (**Table 1**). For example, mice injected with cerulean to induce acute pancreatic inflammation, display an increase in TRPV1 excitability and mRNA expression on NG and DRG neurons (126). Cerulean also leads to leukotriene B4 production by acinar pancreas cells, activating TRPV1 on sensory afferents (89). Treatment with capsazepine, a TRPV1 antagonist, was capable of reducing myeloperoxidase activity and histological severity of acute pancreatitis (127). These findings were further corroborated utilizing intraperitoneal injection of AMG 9810, another TRPV1 antagonist, resulting in diminished pain behaviors in mice (126). Similarly, in a the trinitrobenzene sulfonic acid pre-clinical model of chronic pancreatitis TRPV1 mRNA and protein expression is increased, as is capsaicin-induced activation. This model also induces an increase in the proportion of capsaicin sensitive pancreas-specific DRG neurons, as well as the over expression of NGF, artemin, and GDNF (90, 128). The increased expression of NGF and the GDNF artemin may thus activate TRPV1 on intrapancreatic nerves resulting in the release of substance P (SP) and the maintenance of the disease state (129).

**Table 1:** Pro- and anti-inflammatory TRP channel functions in inflammatory conditions.

TRP Channel	Conditions	Pro-/Anti-Inflammatory	Functionality	References
TRPV1	Pancreatitis	Pro-	Increases histological damage and release of SP, triggering nociception	(89–90)
	Pulmonary inflammation	Pro-/Anti-	CGRP and SP release induces bronchial constriction and elicits coughing reflex/Activation decreases allergic airway inflammation	(24, 91–92)
	Lung Injury	Anti-	Somatostatin is released to diminish neurogenic inflammation and appears to reduce bronchial hypersensitivity	(93, 94)
	Atopic Dermatitis	Pro-	Contributes to itching sensation and dermatitis clinical severity	(95–96)
	Arthritis	Pro-	Upregulation of pro-inflammatory cytokine release, knee joint swelling, and thermal hyperalgesia	(97)
	Sepsis	Anti-	Upregulates anti-inflammatory IL-10 and attenuation of pro-inflammatory CGRP, TNF, and IL-6	(98–100)
	Carditis/Ischemic Injury	Anti-	SP release which increases IL-10 and reduces TNF levels, ROS, and neutrophil infiltration	(101)
				
TRPV4	Colitis/IBD	Pro-	Release of CGRP and SP in hypotonic and irritant conditions and contributes to mechanical hyperalgesia	(102–103)
	Itch	Pro-	Mediates pruritus through cutaneous application of agonists and serotonin and histamine-dependent itch in sunburn and chronic itch	(104–105)
	Sepsis	Pro-	Inhibition of channel significantly decreased systemic cytokines and maintained endothelial cell function	(9, 106)
				
TRPM3	Thermal Hyperalgesia	Pro-	Produces and augments TRPV1/TRPA1 heat-induced nociception in inflamed tissues and mediates CGRP release	(8, 47–49, 54)
TRPM8	Colitis	Anti-	Suppression of pro-inflammatory cytokine release in colitis model and diminishes TRPV1-mediated CGRP release	(7, 52, 107)
	Chronic Neuropathic Pain	Pro-/Anti-	Reduction of thermal and mechanical hyperalgesia, enhances cold hypersensitivity	(5, 108, 109)
	Asthma	Pro-	Increased pro-inflammatory IL-6 and IL-8 release in bronchial tissue	(110–111)
TRPA1	Headache/migraine	Pro-	Increased vasodilation and release of CGRP and SP producing migraine like behaviors	(112–113)
	Allergic Contact Dermatitis	Pro-	CGRP and SP release produces thermal or mechanical hypersensitivity and activation *via* co-localized G-protein coupled receptors	(4, 114–115)
	Acute Lung Injury	Pro-	Releases of pro-inflammatory neuropeptides (CGRP, SP, NKA), ROS, and triggers a cough reflex	(4, 64, 72, 116)
	Asthma	Pro-	Agonist stimulation can induce asthma, increasing bradykinin and ROS	(86, 117–119)
	Colitis/IBD	Pro-/Anti-	AITC administration induced pro-inflammatory IBD conditions/Diminishes histological damage through CGRP release, decreases pro-inflammatory cytokines and oxidative stress	(3, 4, 120–121)
	Arthritis	Pro-	Increases cold and mechanical hypersensitivity in CFA-induced arthritic models, antagonists reduces cartilage, edema, and SP release in paw	(122–123)
	Sepsis/Endotoxemia	Anti-	Attenuates disease severity through modulating release of cytokines IL-1β and IL-6 in mice and decreases serum TNF	(124–125)

In the airways, enhancement of the coughing reflex in humans is associated with an augmented expression of TRPV1 channels on sensory nerves (91, 130). Inhalation of capsaicin has been linked to an increased cough sensitivity in patients with both asthma and chronic obstructive pulmonary disease (COPD; **Table 1**) (24, 131, 132). Similarly, TRPV1 expression and capsaicin sensitivity was increased in myelinated pulmonary afferents in the rat model of ovalbumin-induced airway inflammation (92). Due to the implication of TRPV1 in the initiation of cough, TRPV1 antagonists have been utilized as a treatment to effectively block this reflex (133–135). It has been proposed that TRPV1 activation on C-type fibers releases SP and calcitonin gene-related peptide (CGRP) neuropeptides to induce neurogenic inflammation and airway smooth muscle contraction, thus activating retinoic acid receptors (RARs) to elicit a cough response (136). In models of bacterial lung infections and pneumonia ablation, of TRPV1-positive nerves increase survival, cytokine induction, and bacterial clearance of Staphylococcus aureus pneumonia from the lungs. TRPV1-positive fibers of the vagus nerve in this model release CGRP inducing immunosuppression (137). In contrast, TRPV1 is protective in the LPS-induced model of lung injury. Depletion of TRPV1 causes increased disease severity, with elevated inflammation and bronchial hypersensitivity. When activated during a LPS-induced model of lung injury TRPV1-positive neurons release somatostatin (SST), which acts to diminish neurogenic inflammation (93). Treatment with TRPV1 agonists have additionally been beneficial in treating the ovalbumin-induced allergic airway inflammation. It is believed that SST and CGRP release decrease neutrophil influx and cytokine release (94).

In patients with inflammatory bowel disease (IBD), TRPV1 immunoreactivity is greatly increased in the colonic nerve fibers (138). In a mouse model of IBD, dextran sulfate sodium (DSS)-consuming mice displayed an increase in pelvic afferent activity in response to capsaicin compared to normal mice (139). In gastro-esophageal reflux disease, patients have displayed increased TRPV1 fiber expression in their inflamed esophagus (140).

Similarly, in the skin, an increase in TRPV1 sensitivity and expression of TRPV1 is found in atopic dermatitis (**Table 1** **;** AD) (95). Phospholipase A2 and 12-Lipoxygenase activation of TRPV1 on histamine-sensitive C nerve fibers have been shown to lead to itching sensation (141). Interleukin 31 (IL-31), an inflammatory cytokine, also induces TRPV1-dependent itch, as cutaneous neurons and DRG neurons co-express the IL-31 receptor and TRPV1. TRPV1 deficient mice also display significantly reduced itching in the presence of IL-31 (96). Furthermore, PAC-14028, a potent TRPV1 antagonist, accelerates skin barrier recovery from tape-stripping-induced damage on hairless mice, as well as in both the *Dermatophagoides farinae* and oxazolone-induced dermatitis models. In addition to the accelerated function, PAC-14028 alleviates IgE increase, mast cell degranulation, scratching behavior, and dermatitis clinical severity (95).

In arthritis, inhibition of TRPV1 has identified it as a potential target for therapeutic interventions. In the complete Freund’s adjuvant (CFA) pre-clinical model of arthritis, capsaicin depletion of TRPV1-positive cells, reduces arthritis severity and depletes neuropeptide levels. The depletion of these cells occurs due to a significant increase in intracellular calcium. TRPV1 responds to this influx by desensitizing itself to capsaicin activation, preventing cytotoxic amounts of calcium ions from entering the cell (142). In TRPV1 knockout (KO) mice, swelling of the knee joint and hyperpermeability were reduced. When TNF is directly injected into the knee joint, TRPV1 KO mice have decreased thermal hyperalgesia and joint swelling (97). Additionally, CFA induced arthritis causes a significant increase in TRPV1 expression on the overall proportion of unmyelinated nerves innervating the paw and on DRG neurons (143, 144).

In systemic inflammatory diseases such as sepsis, TRPV1 has an inconsistent role (**Table 1**) (20, 145). In a rat model of endotoxin induced sepsis, pretreatment with capsaicin increases anti-inflammatory cytokine IL-10 levels, and attenuation of CGRP, TNF, and interleukin 6 (IL-6) cytokines (98). In agreement with these findings blocking TRPV1 with capsazepine increases LPS induced hypotension, and mortality rates (146). TRPV1 KO mice further corroborate this, exhibiting elevated hypotension, hypothermia, cytokine levels, organ dysfunction, and mortality in mice with endotoxemia and polymicrobial sepsis *via* cecal ligation puncture (CLP) (99, 147). These studies suggest that TRPV1’s anti-inflammatory role in sepsis, is in modulating nitric oxide (NO), ROS, and TNF (99, 145). However, a contradictory study has also shown that in the same CLP model of sepsis blocking TRPV1 activity with capsazepine attenuates systemic inflammation, multiple organ damage, and mortality (100). The inconsistency in these studies may be a result of capsazepine’s dual ability to antagonize TRPV1 and agonize TRPA1 at similar concentrations, leading to a profound desensitization of not only TRPA1 but the nociceptive neuron (148).

TRPV1-positive sensory nerves innervating the heart have a beneficial role in cardiac inflammation. Genetic depletion of TRPV1 results in excessive inflammation, left ventricular remodeling, and deteriorated cardiac function after myocardial infarction in mice (149). Administration of a TRPV1 antagonist elevates myocardial damage in isolated wild-type hearts, suggesting that TRPV1 may have a protective effect in ischemia-reperfusion injury, with links to the release of SP (129). In ischemia-reperfusion injury CGRP and SP increase the release of anti-inflammatory IL-10, and reduce TNF level, lowering ROS and neutrophil infiltration (150).

The role of TRPV1 in inflammation is complex, with a dependence on disease and tissue-specific actions. Many preclinical studies utilize total body TRPV1 KO mice to elucidate its role in inflammation. However, given the sizable contrast in these findings, further study into the role of TRPV1 in mediating inflammation would benefit from selective knockdown or optogenetic manipulations in different cell types. It is posited that TRPV1 on sensory neurons plays a pro-nociceptive role in acute tissue injury, but an antinociceptive role in chronic conditions (20). Cell type-specific, particularly neuronal, TRPV1 modulation may prove useful in combating the extensive list of inflammatory diseases.

Despite the need for more comprehensive studies on understanding TRPV1 as a potential therapy, TRPA1 been regarded as a therapeutic target for pain and inflammation since the mid-20^th^ century (101). Clinically, desensitization of TRPV1-expressing sensory nerves using high doses of capsaicin has been utilized as a treatment for patients with disorders such as psoriasis, osteoarthritis, cutaneous allergic reactions, pruritus, and peripheral neuropathy (151–153). Though capsaicin has demonstrated clinical benefits, in the 1990s there was a shift away from desensitization due to a common side effect, an intense burning sensation (154) with a prolonged effective duration (155). TRPV1 competitive antagonists, due to their reversible nature and lack of burning, then began to receive attention as potential anti-inflammatory and analgesic therapies.

Clinical trials of such agents have demonstrated varied results based on the disorder in question. TRPV1 antagonists have failed to show benefits in chronic cough and related disorders, such as COPD (156–158). Furthermore, these antagonists demonstrated no significant benefits for patients with seasonal allergic rhinitis (159). On the other hand, TRPV1 antagonists have recently demonstrated efficacy in molar extraction pain (160), mild‐to‐moderate AD (161), pain/stiffness in knee osteoarthritis (162, 163), and gastroesophageal reflux disease pain (164). Recently completed trials of these compounds examine their usage in patients with rosacea (NCT02583009), seborrheic dermatitis (NCT02749383), and skin pruritus (NCT02565134). Interestingly, many clinical trials utilizing TRPV1 antagonists reported that enrolled patients became hyperthermic after administration (19, 164–166), even to the point of trial termination (167). This significant side effect, related to thermal sensitivity, has stalled clinical studies of TRPV1 antagonists (168, 169).

### TRPV4

Similar to other TRP channels, different inflammatory molecules can affect the expression and signaling of TRPV4. Inflammatory cytokines such as IL-1β and interleukin 17 (IL-17) increase TRPV4 mRNA levels in DRG neurons (170) and NGF increases TRPV4 expression in the urothelium (171). Ischemia increases expression in astrocytes (172), while TNF, high-fat and high-alcohol diet (HFA) induce chronic pancreatitis leading to TRPV4 expression in pancreatic stellate cells (173). In patients with active colitis (**Table 1**), tissue samples indicate significant TRPV4 expression on nerve fibers innervating the outer layers of the colon (38). In addition, the inflammatory skin conditions papulopustular rosacea and phymatous rosacea facial, as well as COPD exhibit increased TRPV4 expression in the skin and lungs, respectively (174, 175).

The role of TRPV4 in inflammation has been extensively linked to the Protease-activated Receptor 2 (**Figure 2**; PAR2). PAR2 agonists activate and sensitize TRPV4 in DRG neurons. During intraplanar injection of PAR2 agonist mechanical hyperalgesia and increased pain sensitivity to TRPV4 agonists 4αPDD and hypotonic solutions is induced. 4αPDD and hypotonic solutions additionally stimulate the release of CGRP and SP, with increased sensitivity by the application of PAR2 agonists. Depletion of TRPV4 ablates this PAR2 agonist-induced mechanical hyperalgesia and sensitization (102). Such TRPV4-dependent sensitization is apparent in DRG neurons innervating the mouse colon (176). In addition, PAR2 activation can induce sustained activation of TRPV4 through the production of endogenous agonists, which are believed to increase the duration of PAR2’s proinflammatory effects. This was confirmed using a PAR2-induced paw edema model. When TRPV4 is genetically deleted, paw edema is significantly reduced (103).

The role of TRPV4 in inflammation has not only been linked to PAR2, but to histamine and serotonin as well. Colonic DRG neurons pretreated with histamine and serotonin increased TRPV4 agonist 4α-Phorbol 12,13-didecanoate (4αPDD) induced neural firing *via* PKC, phospholipase C-beta, mitogen-activated protein kinase kinase, and PLA2-dependent pathways. By blocking TRPV4 using siRNA, visceral hypersensitivity induced by histamine or serotonin is significantly reduced, indicating a histamine- or serotonin-mediated response dependent upon TRPV4 in sensory neurons (177). Both serotonin and histamine have also been associated with the induction and exacerbation of pruritus’ or itch responses. When administering serotonin intradermally, inhibition of TRPV4 through genetic deletion or pharmacologic block, reduced itch behavior significantly in comparison to control mice (104). This work has led to the idea that serotonin-evoked pruritus could be mediated by TRPV4 expressed on DRG neurons. In response to acute administration of histamine, no significant differences were found in itch behavior when TRPV4 is inhibited. However, in a chronic setting, histaminergic pruritogens induced itching behaviors were decreased when TRPV4 is blocked, specifically on keratinocytes. This may indicate TRPV4 has a different role in modulating an itch reflex based on the cell type it is expressed on (178). Additionally, TRPV4 has a direct link to itch *via* agonist application. Subcutaneous injection of GSK1016790A, a TRPV4 agonist for example, induces itching behavior in mice (179). Interestingly, in sunburn, in which itch is a common symptom, TRPV4 expression and proalgesic mediator endothelin-1 are enhanced in both humans and mice. Following sunburn, keratinocyte-specific TRPV4 KOs have decreased IL-6 release, with a decrease in the number of recruited neutrophils and macrophages (105). The mechanism for this release has been shown to involve TRPV4-mediated ATP production, stimulation of the P2Y11 receptor, and results in the release of IL-6 and interleukin 8 (IL-8) through the p38 mitogen-activated protein kinases-nuclear factor-κB signaling pathway (180).

In models of sepsis, inhibition of TRPV4 through genetic deletion or pharmacological block has been shown to be protective. In both LPS and CLP models of sepsis inhibition of TRPV4 significantly decreased systemic cytokines, maintained endothelial cell function, and reduced mortality in mice (9). However, there has been contrasting reports on the role of TRPV4 in sepsis, other studies have shown that inhibition has no significant effect on sepsis pathology (106). This inconsistency was found to be due to the antagonist dosage used. That is, excessively low or excessively high doses of TRPV4 agonists cannot effectively treat sepsis. It is hypothesized that a balance in TRPV4 activation is necessary for optimal improvement in sepsis severity (181).

### TRPM3

TRPM3 is primarily linked to the induction of thermal hyperalgesia, a common tissue-level responses to inflammation. Endogenous TRPM3 agonists, such as the neurosteroid pregnenolone sulfate (PS) lowers the thermal response threshold of TRPM3, allowing activation at temperatures as low as 37°C (**Figure 2**) (47). In inflamed regions, this heat sensitization induces a thermal hyperalgesia commonly seen in inflammation. Injection of CFA is commonly used in models of peripheral inflammation and arthritis, in which thermal hyperalgesia often occurs. The role of TRPM3 role in thermal hyperalgesia has been demonstrated through behavioral studies utilizing TRPM3 KO mice. TRPM3 KO mice exhibit increased latency of withdrawal response to a heat test (47). Pretreatment with primidone, an established TRPM3 inhibitor, produces similar responses to the TRPM3 KO studies by preventing CFA-induced heat sensitization (116). Furthermore, TRPM3-deleted mice do not develop thermal hyperalgesia during inflammation, strengthening the channel’s link to inflammatory pain signaling in response to heat (47).

Interestingly, a recent study investigating the CFA-induced model of peripheral inflammation through hind paw injection, revealed that inflammation significantly upregulated TRPM3 mRNA independent of temperature sensing TRP channels, TRPA1 and TRPV1, in DRG neurons innervating the inflamed tissue (8). Additionally, in this model, it has been demonstrated that TRPM3 can augment the responses of both TRPV1 and TRPA1 (8). When isosakuranetin, a TRPM3 specific agonist, is applied to inflamed cutaneous tissue there is a simultaneous reduction in TRPM3 agonist responsiveness as well as a diminished responsiveness to capsaicin and mustard oil, both well-defined TRPV1- and TRPA1-agonists (8). This identifies a potential new role for TRPM3 in inflammation, with the upregulation of TRPM3 playing a key role in thermal hypersensitization (**Table 1**) of DRG neurons co-expressing TRPV1 and TRPA1. Together this is strong evidence that TRPM3 functions primarily as a heat-induced pain sensor in inflammatory conditions.

Viewing TRPM3 as a potential target for inflammation-associated thermal hyperalgesia, certain plant metabolite flavonoids, such as naringenin, ononetin, isosakuranetin, and FDA approved drugs, such as anticonvulsant primidone, may be viable therapeutic options. Thus far, these molecules have shown promise in blocking heat-dependent and PS-induced outward calcium currents within *in vitro* DRG neurons (116, 182). In behavioral experiments, flavanones, and primidone attenuated TRPM3 activation through PS-intraplantar injection by inducing increasingly latent responses to noxious heat (116, 182).

Endogenous regulation of TRPM3 is largely influenced by phosphoinositides (PIPs), such as phosphatidylinositol 4,5-bisphosphate (PIP2) and less abundant phosphatidylinositol (3,4,5)-trisphosphate (PIP3). Whole-cell, patch-clamp recordings demonstrated that decreasing PIP2 concentrations inhibited TRPM3 activity and can be restored through ATP-dependent re-synthesis of phosphoinositides. The restoration process can be attributed to the synthesis of PIPs, in which the increased surge of cytosolic ATP stimulates kinase activity to synthesize PIPs which stimulate TRPM3 (183). Points of negative TRPM3 regulation through depleting PIP concentrations have been observed to occur through phosphatases, notably PIP 5-phosphatases. Antagonists of TRPM3 downregulate the inflammatory channel through the three major Gs, Gq, and Gi/o-coupled G protein-coupled receptors (GPCR), such as Gq-coupled M1 muscarinic, Gi/o-coupled opioid (μ) gamma-aminobutyric acid B, and Gs-coupled adenosine A2B receptors, have shown direct interaction of the Gβγ subunits with TRPM3 (184). Activation of these receptors with their agonists oxoremorine-M, DAMGO ([D-Ala^2^, *N*-MePhe^4^, Gly-ol]-enkephalin), baclofen, and adenosine, respectively, strongly inhibited TRPM3 PS-evoked calcium response on DRG neurons (183, 185). However, it is important to note that the current studies observing the molecular regulation of TRPM3 measure channel activity primarily through PS stimulation, and the implications of these mechanisms modulating TRPM3 thermosensitivity requires additional investigation.

### TRPM8

The key functionalities of TRPM8 in sensing both innocuous and noxious cold (8–26°C) have been shown to play a major physiological role in inflammation, thermoregulation, itch, and migraines (**Table 1**). Numerous studies examining the role of TRPM8 in inflammatory conditions, such as chronic neuropathic pain, noxious cold, and colitis, have demonstrated an upregulation of TRPM8 expression (7, 50–52). One mechanism by which the upregulation of channel expression affects inflammatory conditions is through mediating the release of inflammatory cytokines. Prolonged cold stress (4°C) triggers an increase in rodent hypothalamic TRPM8 expression and corresponds to the decrease of pro-inflammatory cytokine TNF (52). Evidence suggests cytokine regulation through TRPM8 occurs through its interactions with nuclear factor kappa-light chain-enhancer of activated B cells (NFκB), the nuclear import receptor controlling TNF levels. This mechanism could mediate how TNF levels are decreased in response to cold-stress and menthol (52). TRPM8 can also mediate inflammation through crosstalk with other TRP channels. Numerous studies (6, 7, 186) have exhibited how TRPM8 activation suppresses TRPV1-mediated inflammatory neuropeptide, CGRP, release. TRPA1, which also plays a role in the release of inflammatory neuropeptides and pain hypersensitivity during inflammation, has also been observed to become desensitized to exogenous irritant and agonist AITC on sensory neurons that have been pre-treated with icilin. This was confirmed in TRPA1 KO mice, in which icilin induced neuronal activation of the splanchnic nerve is unchanged when compared to wild type mice (107, 186). Thus, TRPM8 may serve an anti-inflammatory function to balance the pro-inflammatory responses of TRPV1 and TRPA1, mediating chemosensory deactivation and inflammatory neuropeptide release.

In preclinical murine models of colitis, TRPM8 appears to regulate inflammation through direct mediation of inflammatory cytokines. Significant upregulation of TRPM8 expression was observed in both chemically induced colitis within mice (trinitrobenzene sulfonic acid; TNBS– and DSS-treatment) and non-inflamed colonic tissue from Crohn’s disease patients (7). Macroscopically, the simultaneous treatment of TRPM8 agonist icilin with TNBS and DSS appeared to substantially diminish colitis-associated histological damage in comparison to TNBS and DSS treatment alone (7). Profiling the effects of icilin on the cytokine distribution revealed a significant reduction in pro-inflammatory cytokines and chemokines, chemokine (C-X_motif) ligand 1, IL-6, monocyte chemoattracted protein-1, IL-1α, macrophage inflammatory protein-1α, macrophage inflammatory protein-1β, and interleukin 12 p40, which likely contributed to attenuated histological damage (7, 187). In mediating CGRP release of other TRP channels, TRPM8 modulation appears to also play a pro-nociceptive role in colonic mechanosensitivity alongside TRPA1 and TRPV4. Pharmacological blockage using AMTB and genetic KOs of the channel produced significant inhibition of distension-induced CGRP release at high pressure (150 mmHg), whereas TRPA1 and TRPV4 inhibition produced a significant attenuation at a lower pressure (90 mmHg) (63). This suggests that TRPM8 works in concert alongside TRPA1 and TRPV4 in controlling CGRP-mediated, colonic mechanosensitivity, however, signals pain at extreme noxious distension levels. The aforementioned crosstalk with TRPV1 appears to be potentially relevant in TRPM8’s regulation of colitis. Capsaicin activation of TRPV1 is known to significantly elevate CGRP levels within the colon, however, prior activation of TRPM8 has been shown to attenuate this response (7). Additionally, icilin treatment and menthol enemas both attenuate TRPV1-mediated release of CGRP in both healthy colonic tissue stimulated with capsaicin and inflammation-induced release (7, 187).

In neuropathic injury models, there has been evidence that TRPM8 plays a role in decreasing mechanical allodynia and thermal hypersensitivity while simultaneously enhancing cold sensitivity. Behavioral experiments generally demonstrate how neuropathic injury models, such as chronic constriction injury (CCI) of the sciatic nerve or spinal nerve ligation (SNL), contributes to cold hypersensitivity post-treatment (5, 108, 109). An original study posited that cold allodynia after nerve injury occurred independently of TRPM8 activity, as the decrease in TRPM8 mRNA levels post-CCI surgery did not correlate with sustained levels of cold hypersensitivity (108); however, more recent experiments have all noted a positive correlation in elevated TRPM8 expression and cold hypersensitivity. Elevated TRPM8 expression in these studies were detected through western blot and mRNA analysis (5, 108, 109). These discrepancies could be attributed to a lack of functional TRPM8 measurements, mRNA levels do not necessarily represent TRPM8 protein or functional expression. TRPM8 has also been suggested to modulate the reflex sensitization to thermal and mechanical stimuli in nerve injury. Injection or topical application of menthol can trigger nociceptive pain in neuropathically injured rodents but is also known to attenuate thermal hyperalgesia and mechanical allodynia (5). Intrathecal injection of TRPM8 antagonist AMTB produces the opposite effect in CCI rodents, causing an increase in thermal hypersensitivity and a decrease in cold sensitivity (109). As a therapeutic measure, TRPM8 activation or inhibition to produce a satisfactory analgesic response is case-dependent on the type of temperature-induced nociception. It has been proposed that low concentrations of menthol may be appropriate to produce sufficient analgesic responses without evoking pain (108).

As with many TRP channels, TRPM8 does not have only anti-inflammatory properties but pro-inflammatory properties as well. For example, asthmatic patients have an upregulation of TRPM8 expression in bronchial epithelial cells and sputum (110, 188). Unlike the analgesic effects produced in colitis or nerve injury, TRPM8 function in the lungs triggers bronchial inflammation through prolonged cold air inhalation. These responses can be replicated in models of pulmonary cold exposure and menthol treatment, causing a significant increase in IL-6, IL-8, and interleukin 25/thymic stromal lymphopoietin receptor (TSLP) mRNA expression (110, 111).

Notable advances in the inclusion of TRPM8 as a potential therapeutic target have been made recently through both chemical antagonists and molecular inhibition through GPCRs. GPCRs known to mediate TRPM8 functionality (e.g., bradykinin and histamine receptors) have been noted to do so through two mechanisms. Continuous activation of phospholipase C by G-α-q depletes PIP2 concentrations in the membrane, which leads to the inhibition of TRPM8-mediated currents (189, 190). The agonistic effect of PIP2 on TRPM8 has been seen through the restoration of TRPM8 in PIP2-depleted membranes through the addition of an aqueous PIP2 analog and activation through high concentrations of PIP2 at warm temperatures 37°C (190). Conclusions are drawn from the inhibition of TRPM8 through PIP2 depletion may likely serve as a method of desensitization/adaptation to continuous cold stimuli in the environment (190). Direct interactions with the G-α-q subunit are an additional mechanism responsible for TRPM8 thermosensitivity in peripheral sensory neurons (191). G-α-q KOs, lowered the TRPM8 potentiation threshold to higher temperatures and increased TRPM8-dependent firing rates in cold conditions, without interfering with PIP2 hydrolysis, indicating the direct inhibitory activity of the G-α-subunit (191, 192). Additional chimeric experiments of G-α-q revealed effector binding sites directly on TRPM8 affirming the direct inhibitory activity for TRPM8 activation (191, 192).

Certain inflammatory mediators can serve as inhibitors of TRPM8 due to their G-α-q-linked GPCRs. Inflammatory mediators possessing Gαq-linked receptors, such as bradykinin and histamine, have shown to inhibit TRPM8-mediated responses to cold temperatures and enhance heat responses on cold-sensitive peripheral fibers. Notably, this response occurred alongside the inhibition of numerous downstream effector proteins, including PKC and phospholipase C, suggesting the independent inhibition of TRPM8 by activated G-α-q (192). This response was not seen in G-α-q KOs, demonstrating G-α-q as a crucial step in gating TRPM8. Additionally, the presence of activated G-α-q desensitizes TRPM8 to positive regulator PIP2, creating a synergistic mechanism to enhance TRPM8 inhibition *via* PIP2 depletion (193). Depending on the inflammatory context, certain conditions that mediate the release of G-α-q-linked inflammatory chemicals possess the ability to counter regulate the anti-inflammatory functions of TRPM8. Thus, we note that multiple mechanisms exist to regulate TRPM8 activity, however, conditions that trigger G-α-q activity may result in reduced TRPM8-dependent anti-inflammatory responses.

Multiple efforts toward the identification of selective TRPM8 antagonists for treating inflammation, chronic pain, and cold-hypersensitivity have led to potential candidates but none have reached clinical settings. Various molecular candidates (arylglycine derivative, benzothiophene-derived phosphonate esters, benzimidazole variants) have been identified to strongly inhibit TRPM8-mediated currents in *in vitro* calcium flux assays, as well as suppress *in vivo* icilin-induced “wet-dog shaking” behavior in a dose-dependent manner (194–196). Among these small molecule inhibitors, studies relating to the benzimidazole derivatives generated greater than 80% inhibition in neuropathic CCI models for cold allodynia in a dose-dependent manner (195, 196). Another noted side effect related to the application of these antagonists is the lowering of core body temperature. Menthol and icilin treatment naturally raise the core body temperature, and certain antagonists have been shown to cause hypothermia by counteracting TRPM8 activation (197, 198). Overall, current drug candidates have been demonstrated to be potent inhibitors of TRPM8 activity through basic *in vitro* and *in vivo* assays, with potential therapeutic implications in inflammation induced cold-induced nociception and allodynia.

### TRPA1

TRPA1 has been shown to have a prominent role in inflammation, both through its expression which can be modulated by inflammatory mediators, and as a regulator of inflammatory signaling. TNF, a well-defined pro-inflammatory cytokine, upregulates the trafficking of TRPA1 to the cell membrane, increasing its membrane expression in peripheral nerves (85). Additionally, when exposing neuronal and epithelial cell lines to viruses such as rhinovirus, respiratory syncytial virus, and measles, both TRPA1 protein and mRNA expression are upregulated (199, 200). Interestingly, neutralizing IL-6 and IL-8 in viral cocultures with neurons blocks the upregulation of TRPA1, indicating that both IL-6 and IL-8 may also play a direct role in increasing TRPA1 expression. In the cerulean-induced pancreatic inflammation model, TRPA1 expression is upregulated on pancreatic sensory nerves (201). Similarly, TRPA1 expression is increased in CFA and nerve injury models of inflammation (202). In many of these models, the upregulation and activation of TRPA1 is accompanied by the release of the generally pro-inflammatory neuropeptides, CGRP, SP, and neurokinin A (NKA) (57, 203). Activation of TRPA1 on TG nerves *via* environmental irritants contributes to this release of SP and CGRP along with increased meningeal vasodilation, which are implicated in migraine pathophysiology (112, 204). Many TRPA1 agonists that are linked to the release of SP and CGRP have also been shown to induce migraine or headache behaviors (113, 205). Interestingly, TRPA1 agonists have not only demonstrated pro–inflammatory properties, but anti-inflammatory as well depending on location and disease context.

Many TRPA1 agonists have been shown to induce a local inflammatory response when topically applied to skin. AITC, the molecule responsible for the pungent taste of mustard, horseradish, and wasabi, for example induces the release of CGRP and SP, causing thermal and mechanical hypersensitivity, also known as an increased pain sensitivity, associated with inflammation (4). Cinnamaldehyde, the molecule that gives cinnamon its flavor and odor, also induces acute skin inflammation when applied topically. Mice receiving topical cinnamaldehyde exhibit edema formation and dermal leukocyte infiltration (206). Additionally, many contact dermatitis reactions are TRPA1 mediated. Xylene and toluene are common solvents that can induces a significant inflammatory response of edema and pain when exposed to skin. However, when the skin of TRPA1 deficient mice were exposed to xylene or toluene, the inflammatory response was ablated, and when wild-type mice were treated with a TRPA1 antagonist orally (HC-030031) the inflammation of the skin was significant reduced (207).

As previously mentioned, itch is a common symptom of skin irritation and dermal inflammation associated with different conditions, such as allergic contact dermatitis (**Table 1**; ACD), AD, and psoriasis. Itch warns against harmful environmental irritant, and the urge to scratch is an evolutionary mechanism to remove irritants from the affected area, characterized by swelling and infiltration of immune cells such as lymphocytes (114). In histamine-independent itch, it has been found that itch related GPCRs activate TRPA1 to initiate a local inflammatory response. These itch GPCRs (G protein-coupled bile acid receptor 1, TSLP, MAS-related G protein coupled member A3, and MAS-related G protein coupled member C11) co-localize with TRPA1 on cutaneous sensory nerves (115, 208). In ACD, a major obstacle is histamine-independent inflammation. ACD is a common inflammatory skin condition caused by hypersensitivity to allergens (114, 209). In both acute and chronic models of murine contact dermatitis, symptoms of ACD were significantly decreased with both pharmacological inhibition as well as genetic ablation of TRPA1. When inhibiting TRPA1, it was found that local levels of proinflammatory cytokines interleukin 4, IL-6, and chemokine (c-x-c motif) ligand 2 were decreased along with dermatitis scores, edema, swelling, and T cell infiltration (209). In psoriasis ROS and RNS play a critical role in its pathology inducing oxidative and nitrosative stress activating TRPA1 channels on sensory nerves innervating the skin, causing the release of SP and CGRP. In a preclinical model of psoriasis, TRPA1 antagonists significantly inhibited itching, however it Is important to note that long term treatment with a TRPA1 antagonist or TRPA1 deletion is actually associated in increased psoriasis skin phenotype (210).The differences in long term vs. short term treatment may be linked to TRPA1 on other cell types, as additional studies have shown a role for TRPA1 on immune cells (71, 210–212).

Similar to its role in dermal inflammation, TRPA1 plays a key role in pulmonary inflammation. TRPA1 is expressed on afferent vagus neurons, specifically located on Aδ-, and C-fibers, which densely innervate the lungs. Noxious irritants in the air such as heavy metals, general anesthetics, cigarette smoke, and tear gas have been shown to activate TRPA1. Once activated, these TRPA1-positive neurons locally release of CGRP, SP, and NKA to induce inflammation, bronchoconstriction, vasodilation, and infiltration of immune cells (4, 64, 72). In addition, pulmonary TRPA1 stimulation can induce airway reflex responses such as coughing (117). Experimentally, multiple exogenous TRPA1 agonists have been used in animal and human models of cough such as, citric acid, cinnamaldehyde, and AITC. These responses are dose dependent and significantly reduced with the inhalation of TRPA1 antagonist HC-030031 (117, 213, 214). Endogenous TRPA1 agonists are also known induce cough, which occurs in response to tissue inflammation in conjunction with diseases such as asthma.

In asthmatic patients, the airway is hyperreactive and can cause bronchoconstriction. Many TRPA1 agonists listed above can induce asthma, such as cigarette smoke and the leading cause of occupational asthma, toluene diisocyanate is a strong TRPA1 stimulant. Endogenous activators of TRPA1 have also been shown to induce asthma. Asthmatic lungs exhibit increases in bradykinin, 4-hydroxynonenal and ROS, which are all TRPA1 agonists. This increase in ROS in turn leads to elevated oxidative stress (86, 117, 215–217). In murine and rat, ovalbumin-induced models of asthma, a decrease in late asthma response symptoms is seen post TRPA1 antagonist treatment (118). In TRPA1-deleted mice, airway infiltration of leukocytes is significantly reduced, as are the levels proinflammatory cytokines interleukin 5, interleukin 13, and TNF (4, 118, 119, 217).

Unlike its role in dermal and pulmonary inflammation, TRPA1 has been found to have contradictory pro- and anti-inflammatory roles in gastrointestinal inflammation. TRPA1 is widely expressed in the gut, with functional expression found on neurons innervating the intestine and mucosal endocrine cells. In patients with Crohn’s disease (CD) and ulcerative colitis (UC), as well as preclinical models of IBD TRPA1 expression is upregulated in colonic tissue (3). In animal models of colitis, TRPA1 has an inconsistent role inflammation. For example, genetic deletion of TRPA1 has been found to both decrease and aggravate the disease (120, 212, 218), while blocking TRPA1 with an antagonist and activating with an agonist can be protective (120, 218). These contradictory occurrences may be due the opposing inflammatory effects of GCRP and SP in the gut. Activation of TRPA1 on afferent nerve fibers innervating the colon as well as DRG and NG neurons induce the release both CGRP and SP. However, in the gut the role of CGRP in inflammation is reversed compared to other locations of inflammation. In experimental models of colitis, it is widely accepted that CGRP is protective and a lack of CGRP increases the susceptibility to spontaneous colitis, as well as experimentally induced colin damage. A local block of the receptor for CGRP, calcitonin receptor-like receptor, also increases colitis severity (4, 10, 121, 219). Different IBD models as well as the stage of the disease could also be the source of these contradictions, AITC induced IBD, indicates TRPA1 has having pro-inflammatory properties, as does the TNBS induced model of colitis (3, 10, 220, 221). The DSS-induced model of colitis has also shown contrasting results, with TRPA1 genetic deletion or treatment with antagonists increasing disease severity in some cases, but alleviating disease severity in others (212, 221). In addition, treatment with cannabiderol and cannabidivarin, both non-psychotropic cannabinoids, and TRPA1 agonists have shown anti-inflammatory effects, decreasing disease severity in the dinitrobenzene sulfonic acid model of IBD (3, 222, 223). Other TRPA1 agonists have also found to be anti-inflammatory when used to treat intestinal inflammation. Carvacrol and carvacryl acetate were used as effective treatments of intestinal inflammation, decreasing levels of pro-inflammatory cytokines, and oxidative stress. Blocking carvacrol and carvacryl acetate treatment with a TRPA1 antagonist, reversed these beneficial effects (224, 225). Overall, TRPA1 is an interesting target for treating gastrointestinal inflammation, however, more studies are needed to fully understand its role in this context.

In models of arthritis, TRPA1 has been linked both to joint inflammation and hyperalgesia (**Table 1**). In many studies using monoiodoacetate and CFA models of arthritis, genetic depletion of TRPA1 or antagonist inhibition, have resulted in significant reduction in cold and mechanical hypersensitivity (122, 123, 226–228). In arthritis-induced joint edema, erosion, and inflammation, TRPA1 does not have a well-defined role, with conflicting results reported. In some studies, TRPA1 was not found to have a prominent role in knee joint swelling or paw edema as genetically deleting TRPA1 and blocking with an antagonist had no effect on TNF and CFA induced joint swelling (122). In contrast, other studies have also shown that either genetic deletion or blocking of TRPA1 reduces changes in cartilage, edema, and SP release in the paw (123, 227–229). Although the extent of the TRPA1 channel contribution to arthritis is not conclusive, the current evidence shows the potential for further study to understand and potentially target TRPA1 as an arthritis therapy.

Unlike the previous inflammatory disease models, with localized regions of inflammation, models of systemic inflammation have shown TRPA1 to be generally protective. Treatment with TRPA1 agonists, cinnamaldehyde, AITC, fentamate non-steroidal anti-inflammatory drugs, and carnosol have all been shown to be protective in inflammation. In the CLP model of sepsis, blocking TRPA1 increases disease severity as well as the levels cytokines IL-1β and IL-6 in mice (**Table 1**) (124). In an LPS induced model of inflammation, oral administration of cinnamaldehyde deceases NO, TNF, high mobility group box protein 1, interleukin 18 and other inflammatory mediators in serum and plasma (230, 231). Cinnamaldehyde can also attenuate apoptosis and promote neuronal survival in DRGs due to oxidative stress, by inhibiting NFκB and decreasing ROS (232). Furthermore, direct activation of TRPA1 on the cervical vagus nerve by the optopharmacological molecule optovin reduces systemic inflammation induced by LPS by significantly decreasing serum TNF (125). Interestingly, LPS itself has recently been found to directly activate TRPA1 directly on sensory neurons, inducing a calcium influx, along with vasodilation, pain, and the release of CGRP in a TRPA1 dependent manner (73).

Based on many of the preclinical discoveries previously mentioned, TRPA1 has been increasingly identified as a potential therapeutic target for pain and inflammation. Between 2015 and 2019, twenty-eight patent applications were filed for TRPA1 antagonists, many of which were aimed at treating pain, airway respiratory diseases, and dermatological disorders. Currently, to our knowledge, only five of the twenty-eight patented molecules have gone on to clinical trials, with only a few making it to phase II and none reaching phase III. Translation of TRPA1 antagonists from pre-clinical to clinical results remains challenging, some have shown strong activity in humans with reduced or no antagonist activity in mice or rats. Additionally, as TRPA1 is expressed in a wide variety of tissues with varying roles in inflammation, this makes it difficult to determine safety and efficacy without very targeted approaches (233, 234). Current preclinical models lack tissue specificity for *in vivo* modeling. Similar to TRPV1 preclinical studies, many preclinical models utilize whole-body genetic deletion when evaluating the role of TRPA1 in inflammation. Additional studies looking into tissue-specific genetic modification and activation would greatly benefit in identifying the potential role of TRPA1 as a therapeutic target in inflammation. Despite these challenges, TRPA1 remains a key target for treating inflammation with the potential for more disease and tissue-specific targeted therapies.

## Conclusions

The broad selectivity and polymodal nature of TRP channels make them critically important in sensory transduction and integration. Their presence on various immune cells (e.g., macrophages, T-cells) and wide distribution across sensory nerves (**Figure 1**) suggests that they may be important conduits for neural-immune crosstalk. Moreover, because their activation often leads to the release of neuropeptides linked to neurogenic inflammation (**Figure 2**), their expression on peripheral nerve and on epithelial cells may be key factor in a wide range of conditions and inflammatory disorders that we have discussed in this review including sepsis, arthritis, asthma, colitis, pain, and dermatitis (**Table 1**).

Interestingly, there is some discrepancy with the understanding of TRP channels role in many of these inflammatory disorders. Both role and specificity have been disputed which could be influenced by multiple variables. First, in preclinical disease models, the availability of multiple different models for an individual disease adds to the variability of TRP channel contributions in different disease contexts. Second, there is a lack of appropriate genetic models for many of these diseases. Given the broad range of tissue expression and polymodal activation of TRP channels, utilizing whole-body genetic deletion models often results in differing outcomes, when compared to cell-type or tissue-specific genetic manipulations. As observed with TRPV1 and TRPA1, the site of the inflammation in addition to how widespread it is (local vs. systemic) influences its pro- or anti-inflammatory function (**Table 1**). Cell and region-specific KO models would greatly enhance our understanding of how TRP channels modulate inflammation, which would additionally allow for better targeted therapeutics. In addition, as TRP channels are activated by a wide range of agonists, both exogenous and endogenous, it is important to understand how inflammatory responses differ depending on disease states and depending on the location within the body. An emerging area of interest within TRP channel research is focused on how the nervous system deciphers immune-related signals to initiate, trigger, and modulate inflammation. TRP channels, including those discussed in this review, play an important role in this communication between the nervous and immune systems. As the field advances to discover more about the mechanisms and roles of TRPV, TRPM, and TRPA channels, it will be exciting to see if targeted drug development based on a better mechanistic understanding can provide therapies to treat the assortment of conditions involving TRP channels.

## Author Contributions

HS, NK, AC, SC, and EC wrote and edited the manuscript. All authors contributed to the article and approved the submitted version.

## Funding

This work was supported, in part, by NIH 5R01GM132672 (SC).

## Conflict of Interest

The authors declare that the research was conducted in the absence of any commercial or financial relationships that could be construed as a potential conflict of interest.

## Abbreviations

AITC, allyl isothiocyanate; CFA, complete Freund’s adjuvant; CGRP, calcitonin gene-related peptide; IBD, inflammatory bowel disease; IL-6, interleukin-6; IL-8, interleukin-8; IL-10, interleukin-10; NKA, neurokinin A; ROS, reactive oxygen species; SP, substance P; TNF, tumor necrosis factor.
